# The fate of visual long term memories for images across weeks in adults and children

**DOI:** 10.1038/s41598-022-26002-7

**Published:** 2022-12-16

**Authors:** Annabelle Goujon, Fabien Mathy, Simon Thorpe

**Affiliations:** 1 Laboratoire de Recherches Intégratives en Neurosciences et Psychologie Cognitive UR 481, Université de Franche-Comté, 19 rue Ambroise Paré, 25030 Besançon, Cedex, France; 2 Laboratory BCL CNRS UMR 7320 & Université Côte d’Azur, Nice, France; 3 CerCo-CNRS & Université de Toulouse 3, Toulouse, France

**Keywords:** Neuroscience, Psychology

## Abstract

What is the content and the format of visual memories in Long Term Memory (LTM)? Is it similar in adults and children? To address these issues, we investigated, in both adults and 9-year-old children, how visual LTM is affected over time and whether visual vs semantic features are affected differentially. In a learning phase, participants were exposed to hundreds of meaningless and meaningful images presented once or twice for either 120 ms or 1920 ms. Memory was assessed using a recognition task either immediately after learning or after a delay of three or six weeks. The results suggest that multiple and extended exposures are crucial for retaining an image for several weeks. Although a benefit was observed in the meaningful condition when memory was assessed immediately after learning, this benefit tended to disappear over weeks, especially when the images were presented twice for 1920 ms. This pattern was observed for both adults and children. Together, the results call into question the dominant models of LTM for images: although semantic information enhances the encoding & maintaining of images in LTM when assessed immediately, this seems not critical for LTM over weeks.

## Introduction

How are the landscapes of your last trip, the layout of the bedroom in which you grew up, the face of your teacher when you were eight years old seared into your memory? How are images from unique visual episodes encoded, then consolidated to emerge as memories or recycled in the construction of new percepts? Studying the formation and the consolidation of sensory memories raises the problem of the content and format of such memories in Long Term Memory (LTM). In this respect, the present study aimed at investigating how visual LTM is affected by time and whether visual features vs semantic/conceptual information in visual LTM are affected differently over weeks. This question was examined in both adults and children.

In a closely related field, the literature on mental imagery has traditionally opposed two main classes of hypotheses to account for the coding of images in LTM. The first refers to the propositional position, which assumes that symbolic codes are used for LTM (for reviews^1,2^). These codes represent something conceptual and sometimes arbitrary as opposed to perceptual. In this view, coding in memory would be a sentence-like description of the image. By contrast, the functional-equivalency hypothesis supposes that the coding of images in memory has the same structure as the information being represented^3–5^. In this view, symbolic codes are not required to account for LTM. At the interface, the dual-code theory assumes that both analogue (or perceptual codes), and arbitrary symbols or verbal codes are used when retrieving representations of pictures from memory^6,7^.

Questions about the content and format of visual memories have also been addressed in the field of the perception of visual scenes through research aimed at assessing both the capacity of visual LTM and the fidelity of our representations of visual stimuli. In the 1960s and 70s, research using large scale memory procedures revealed that people have an extraordinary capacity to remember thousands of images presented for only a few seconds each^8,9^. These studies concluded that the number of visual items that can be stored in LTM is potentially unlimited, that such memories last for at least several days, and that memory performance depends primarily on the distinctiveness between the target stimulus and the concurrent stimulus (foil stimulus) in the memory task (e.g., recognition)^10^. Nonetheless, because of the substantial visual and semantic heterogeneity between the used stimuli, those studies did not provide relevant information regarding the coding of visual memories into LTM.

Three decades later, this issue received renewed interest following research reporting the phenomena of change blindness and inattentional blindness^11^. The dramatic inability to detect even massive changes in the visual input led many authors to claim that memory representations for real-world stimuli are impoverished, sparse, volatile and lack visual details^12–16^. Influential theories in the early 2000s postulated that representations in visual LTM are gist-like and semantic in nature (e.g.^17^). This position was later examined and undermined. The ability of participants to detect changes when they are tested with either forced-choice paradigms or with longer exposures provided strong evidence that visual episodes leave a more complete memory trace that includes “visual” (or perceptual) information and not just the gist^18^. Large scale memory studies have subsequently strongly supported this conclusion, showing the massive capacity to store visual details from objects or scenes in visual LTM (for reviews^19,20^). For instance, participants initially exposed to 2500 objects for 3 seconds performed at 92 % in a two-forced choice recognition task when the target and the foil stimulus belonged to a different category, 88% when they belonged to the same basic-level category and 87% when the same object was presented in a different state or pose^21^.

Recent research aimed at determining what makes an image memorable suggests, nonetheless, that high-level properties, such as distinctiveness, atypicality, emotional valence and semantic attributes strongly contribute to its memorability. In contrast, low-level image properties, such as the salience, color or other simple image features make relatively weak contributions^22–24^. While objects without semantics might not be effective at predicting memorability, the presence of semantic labels associated with objects or photographs could improve it. For example, the possibility to provide a single label for each image (i.e. a single gist) might explain most of what makes an image memorable^22^. Scene semantics would therefore be a primary substrate of memorability.

Thus far, most models and theories of VLTM give more weight to conceptual features than perceptual features in the coding used to retrieve visual representations in memory^25–29^. “*Being perceptually rich and distinctive might be not sufficient to support VLTM*. (…) *VLTM representations are hierarchically structured, with conceptual or category specific features at the top of the hierarchy and perceptual or more category-general features at lower levels of the hierarchy”* (Brady et al., 2011, p19^19^). According to Mary Potter (2012a, p1^28^), “*although some specific visual information persists, the form and content of the perceptual and memory representations of pictures over time indicate that conceptual information is extracted early and determines most of what remains in LTM”.*

However, in most studies on visual LTM, the contents of memory were examined either immediately after learning or the next day. Thus, the question of how memories for images evolve over time remains unanswered. Yet this issue is crucial to determine how visual representations are transformed and consolidated into visual memories. In this framework, the goal of this study was to examine how visual and semantic features were affected by delays and to test whether the hypothesis according to which “*conceptual information is extracted early and determines most of what remains in LTM”* extends to memories that persist beyond several weeks. This hypothesis was examined in both adults and nine-year-old children.

The literature on memory development across the life span suggests large developmental differences in many aspects of memory, especially working memory^30^ and declarative memory^31,32^. Nonetheless, visual recognition memory is usually thought to be an early emerging form of memory, which can be measured from the first months of life^33^. Using an abbreviated version of the materials developed by Brady et al. (2008), Ferrara, Furlong, Park, and Landau^34^ reported impressive visual memory performance by four-year-old children, both in terms of the large number of items and the level of details required for recognition. Although the number of images was substantially less than in the experiments conducted in adults, the patterns of results were similar. However, to our knowledge very few studies, if any, have examined how memory for images evolved over weeks and whether this evolution differed across the development.

**Figure 1:**
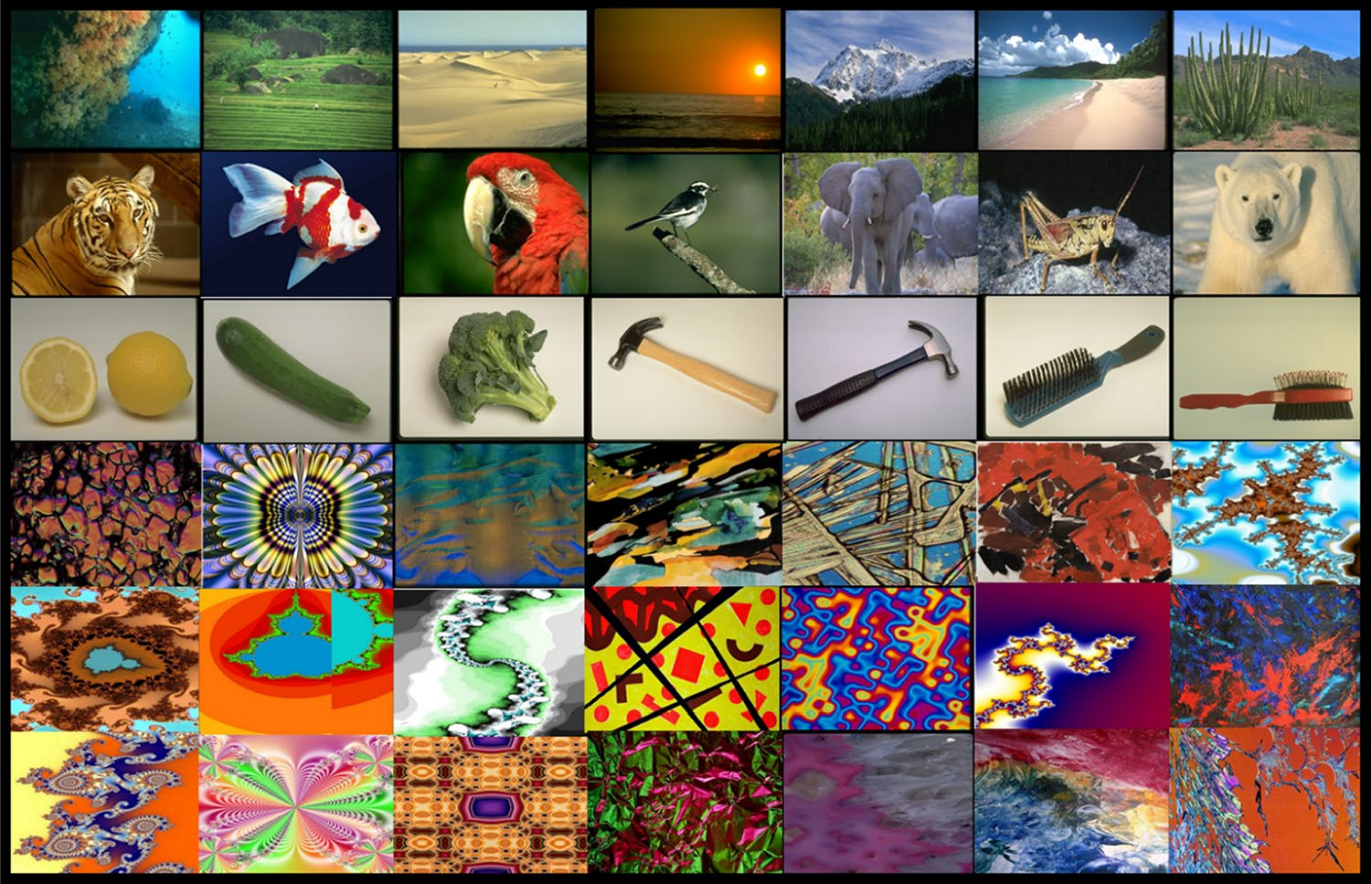
Examples of meaningful images (top three rows) and meaningless images (bottom three rows) used in the experiment. The images came from the CerCo lab’s collection of images.

In this framework, we investigated, in both adults and nine-year-old children, how the recognition of images evolves over time, depending on whether they were meaningful or meaningless (Fig. 1). The meaningful images were photographs of real-world scenes or objects. They were supposed to be easy to label (i.e. the gist was supposed to be automatically extracted). The meaningless images were abstract paintings, fractal images, or complex geometrical figures and were supposed to have no meaning *a priori*. This assumption has been validated in a pilot experiment. In this experiment, participants had to give a single label to those images presented once or twice during a learning phase. The results showed that for the meaningful images presented twice, the participants provided the same single label in 85 % of the time. In reverse, they had much more difficulty to provide a label to the meaningless images and this label was consistent between the two exposures in only 35% of the time. Moreover, this label was mostly related to the global colored pattern of the image and a same label was used for many different images. Thus, in the framework of this study, we considered as meaningful, images that could be designated with a single label (i.e. a single gist), and as meaningless, images that were not derived from real-world, and for which the gist is not given *a priori*, and not extracted automatically.

The experiment included two phases. In a learning phase, participants were exposed to hundreds of meaningless and meaningful images. Because most models on visual memory have been based on research using *Rapid Serial Visual Presentation* (RSVP) procedures or *large scale memory* procedures (for examples that combines both procedures, see^35,36^), two exposure durations were examined. Indeed, based on this literature, exposure duration seems to have different impact on memory performance and specifically on the extraction of visual vs. semantic features. Thus, change blindness might be due to a lack of encoding time or attention to each object instead of memory limitations for visual details^37^. Because we assumed a strong impact of the duration, the images were presented for either 120 ms or 1920 ms during the learning phase. We also examined the impact of another factor that potentially plays a critical role in memorization, that is, the repetition of the images. Indeed, we expected that a single exposure might not be sufficient to maintain an image for a very long term in memory. Thus, the images were presented either once or twice during the learning phase.

**Figure 2:**
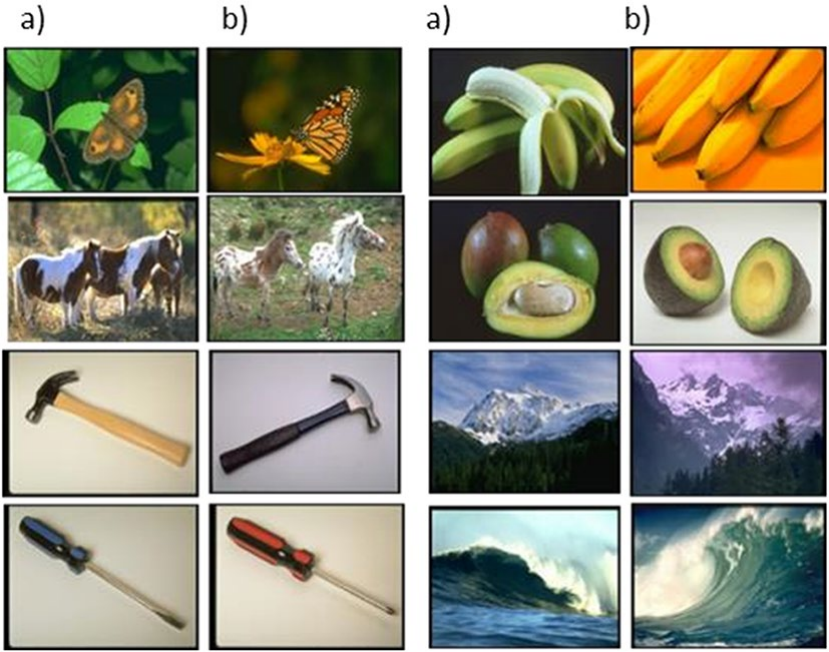
Examples of images used for the exemplar condition. For example, the images (**a**) were presented during the learning phase and the images (**b**) during the testing phase. The images came from the CerCo lab’s collection of images.

Immediately after the learning phase, or after a delay of three weeks or six weeks, the memory of the participants was assessed through a recognition task that included old and new meaningless and meaningful images. Among the new meaningful images, some belonged to a basic-level category not used during the learning phase (novel images), and some belonged to a basic-level category that had already been used during the learning phase (exemplar lures). This is illustrated in Fig. 2.

Participants were first asked to judge whether the image was old or new and then to indicate how confident they were in their response using a 4-point confidence scale (“Confidence? 1= just guessing, 2 = not sure, 3 = confident, 4 = very sure). Collecting those confidence ratings aimed at determining the most relevant measure to compare meaningful and meaningless conditions, given potentially different response biases in the meaningful and meaningless conditions^38^. An examination of receiver operating characteristic curves (ROC), derived from signal detection theory (SDT) should help to provide the best model to apply to our data^39^.

The hypothesis that semantic information is extracted earlier and determines most of what remains in LTM^28^ leads to four predictions: (1) For very brief exposures, only meaningful images should be accessible to recognition; (2) Meaningless images should be more subject to forgetting over weeks than meaningful images; (3) False recognition for the exemplar lures should be more numerous than false recognition for novel images, and this effect should increase over time. Indeed, if only the gist is retained across weeks, more and more confusion between the old images and the exemplar lure images should be observed. (4) Concerning the developmental aspects, we expected lower performance in children. Nevertheless, in view of the literature on children visual memory, similar patterns of results might be observed in nine-year-old children and in adults^33,34^. Given the weakness of the literature in the field, this question remains nevertheless very exploratory.

## Results and discussion

**Figure 3:**
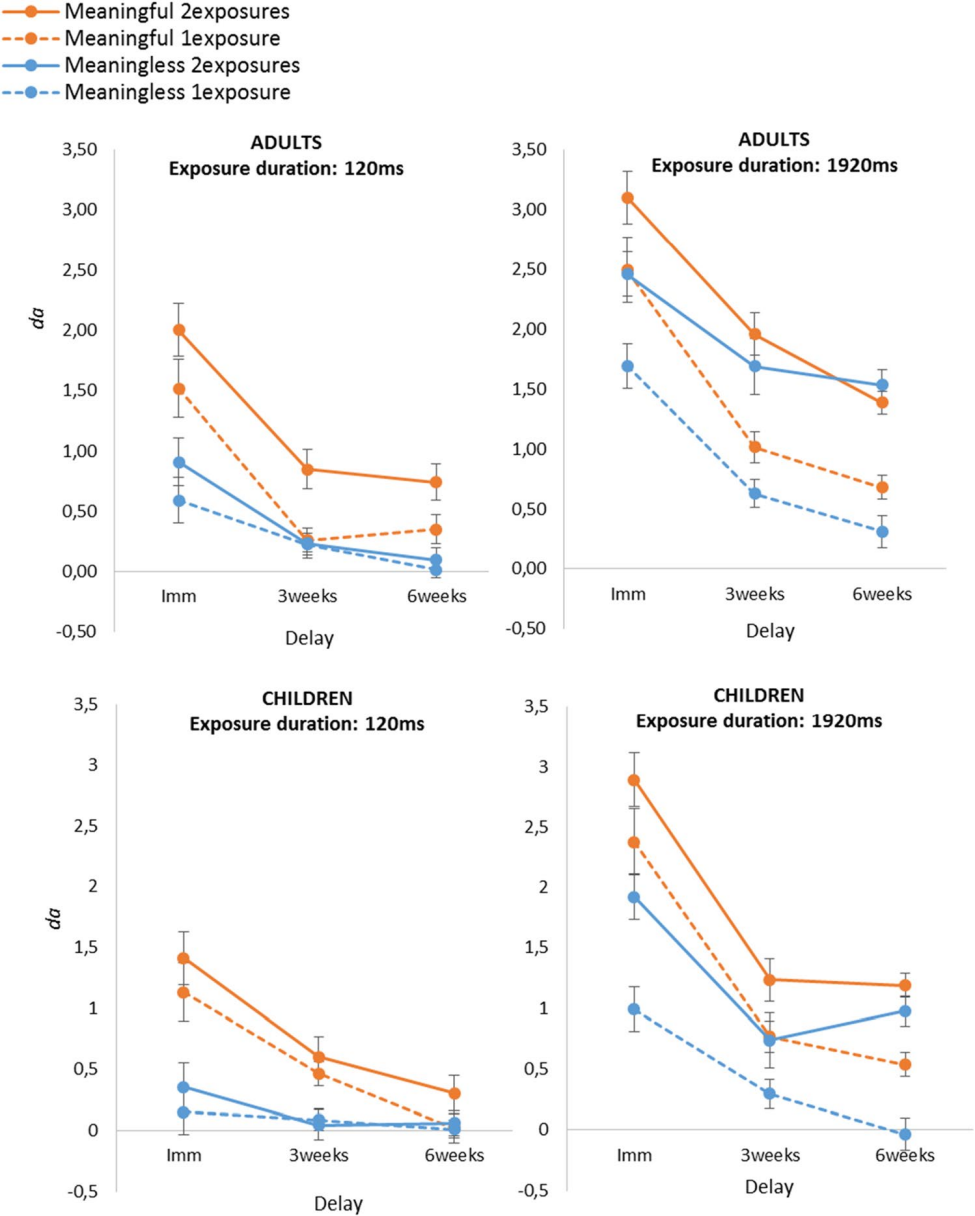
Mean discrimination indexes (*d*_*a*_) depending on the delay (immediate, 3-weeks, and 6-weeks), the type of images (meaningful vs. meaningless), the exposure duration (120 ms vs. 1920 ms) and the number of exposures (1 vs. 2). The top panels display the *d*_*a*_ observed in adults and the bottom panels display the *d*_*a*_ observed in children. The error bars show the standard error of the mean (n = 12).

The hits (i.e., when the image is old and the participant's response is old) and the false alarms (FA, i.e., when the image is new and the participant's response is old) observed in the recognition task depending on the type of images, the exposure duration (120 vs. 1920 ms), the number of exposures (1 vs. 2), the delay (immediate vs. 3-weeks vs. 6-weeks) and the age of participants (adults vs. children) are shown in Supplementary materials, Tables S1 & S2. The ROC curves in each condition derived from the confidence ratings are also shown in Supplementary materials, Figs. S1 & S2. Examination of the zROC (which corresponds to z scores of hits and FA plotted as coordinates) revealed a slope almost always different than 1, suggesting Gaussian distributions of unequal variance in the participants’ responses. Therefore, recognition accuracy was calculated using the discriminability measure of *d*_*a*_^38^. Each *d*_*a*_ was computed separately from the false-alarm and hit rates for each participant, for each type of image (meaningless vs. meaningful) and exposure condition (120 vs. 1920 ms and 1 vs. 2 exposures). Each *d*_*a*_ was also corrected by the slope of the zROC in each condition. The *d*_*a*_ was calculated as follows:

$$d_{a} = \left( {\frac{2}{{1 + s^{2} }}} \right)^{1/2} \left( {z_{H} - sz_{F} } \right)$$

where *s* corresponds to the zROC slope, *Z*_*H*_ to the z-scores of the hits and *Z*_*F*_ to the z-scores on the FA. The *d*_*a*_ values are shown Fig. 3.

### Analysis on ***d***_***a***_ as a function of Age * Delay * Type of images * exposure Duration * Number of exposures

To compare how memory for meaningless vs. meaningful images evolved over weeks, we carried out a mixed-design analysis of variance on *d*_*a*_ with Age (adults and children) and Delay (immediate, 3-weeks, and 6-weeks) as between-subject factors, and Type of images (meaningful vs. meaningless), exposure Duration (1920 ms vs. 120 ms) and Number of exposures (1 vs. 2) as within-subject factors. Because we ran a multiway anova which could lead to unexpected interactions, we decided to apply a correction to *p*-values as recommended^40^. Indeed, running a multiway analysis of variance harbors a multiple-comparison problem. In the case of five factors, there are 31 effects to be tested (i.e., 5 main effects, 10 first-order interactions, 10 second-order interactions, 5 third-way interactions, and 1 four-way interaction). To control for the familywise error rate, we therefore applied a Bonferroni correction to set a more conservative *p*-value (*p* = 0.05/31 = 0.0016).

The analysis revealed a main effect of each factor: Age, *F*(1,66) = 11.15, *p* = .001, η_p_^2^ = 0.14; Delay, *F*(2,66) = 48.21, *p* < 0.001, η_p_^2^ = 0.59; Type of images; *F*(1,66) = 144.25, *p* < 0.001, η_p_^2^ = 0.69; exposure Duration, *F*(1,66) = 422.91, *p* < 0.001, η_p_^2^ = 0.87; Number of exposure, *F*(1,66) = 231.89, *p* < 0.001, η_p_^2^ = 0.78. Those results show that, (1) adults had better memory performance than children; (2) memory for images decayed strongly over weeks; (3) memory for meaningful images was better than memory for meaningless images; (4) memory benefited from multiple and extended exposures.

There were significant interactions [Number * Duration, *F*(1,66) = 80.44, *p* < 0.001, η_p_^2^ = 0.55] and [Duration * Delay, *F*(1,66) = 21.73, *p* < 0.001, η_p_^2^ = 0.40], suggesting that multiple and extended exposures had a beneficial effect on memory and that the exposure duration delayed the forgetting in memory. More crucially, the results yielded significant interactions between the factors [Type of images * Delay, *F*(2,66) = 22.26, *p* < 0.001, η_p_^2^ = 0.40] and [Type * Duration * Number, *F*(1,66) = 19.83, *p* < 0.001, η_p_^2^ = 0.23]. These findings suggest that the meaningless images were less affected than the meaningful images by the factor delay, and that the meaningless images benefited more from a second and longer exposure.

The other interactions were not reliable using the corrected significant level *p* = 0.0016, [Number * Age, *F*(1,66) = 7.25, *p* = 0.009, η_p_^2^ = 0.10], [Duration * Age * Delay, *F*(1,66) = 4.07, *p* = 0.02, η_p_^2^ = 0.11], [Type * Duration * Age, *F*(2,66) = 6.40, *p* = 0.01, η_p_^2^ = 0.09], [Duration * Age, *F*(1,66) = 3.83, *p* = 0.05, η_p_^2^ = 0.06], [Number * Age * Delay, *F*(2,66) = 2.75, *p* = 0.07, η_p_^2^ = 0.08], [Type * Age, *F*(1,66) = 1.63, *p* = 0.21, η_p_^2^ = 0.02], [Type * Number * Delay, *F*(1,66) = 2.23, *p* = 0.17, η_p_^2^ = 0.06], [Type * Duration * Delay * Age, *F*(1,66) = 1.73, *p* = 0.18, η_p_^2^ = 0.05], [Type * Duration * Number * Delay * Age, *F*(1,66) = 1.13, *p* = 0.33, η_p_^2^ = 0.03], [Number * Duration * Delay, *F*(1,66) = 2.56, *p* = 0.085, η_p_^2^ = 0.07], [Number * Duration * Age * Delay, *F*(2,66) = 1.20, *p* = 0.308, η_p_^2^ = 0.03] (for all others interactions, *Fs* < 1).

We then ran a few Bayesian analyses to more firmly conclude about the robustness of the respective effects, but also to compare children and adults. In these analyses, reported in supplementary materials, we found evidence for the effects and interactions revealed by the ANOVA described above, and confirmed that although memory performance was generally weaker in children, the patterns of performance across weeks were not significantly different between both populations, with nevertheless anecdotal interactions with the factor Age (see supplementary materials).

### Post-hoc comparisons with Type * Delay * Age

Our main result so far concerns the interaction between the factors Type of images and Delay (interaction strongly confirmed by the Bayesian analyses). Indeed, we expected an interaction, but in the opposite direction. Recall that we expected that meaningless images would be more forgotten over weeks than meaningful images. Conversely, the results showed that the meaningless images were less affected than the meaningful images by the factor delay. To refine the analysis, we conducted post-hoc comparisons with the factors Type and Delay and with the additional factor Age. Those comparisons revealed that in adults, memory for the meaningful images was better when assessed immediately (*t* = 7.63, *P*_*holm*_ < 0.001). But this benefit was no longer present at 3-weeks (*t* = 2.87, *P*_*holm*_ = 0.180); and 6-weeks (*t* = 2.64, *P*_*holm*_ = 0.286). In children, a benefit for the meaningful images was observed when memory was assessed both immediately (*t* = 9.78, *P*_*holm*_ < 0.001) and three weeks after learning (*t* = 4.21, *P*_*holm*_ = 0.003), but the benefit disappeared after six weeks (*t* = 2.28, *P*_*holm*_ = 0.588).

### ANOVA confined to the condition “2 exposures–1920 ms”

To ensure that the interaction Type x Delay was not due to combination of floor effects across weeks in the conditions “one exposure” and/or “120 ms” that could lead to a Type II error, we conducted a mixed-design ANOVA confined to the most favorable condition, that is, the combined condition “2 exposures–1920 ms”, with the factors Type, Age and Delay. Indeed, this unique condition of exposure seems to be required to maintain meaningless and meaningful images in memory for six weeks, in both adults and children. After applying the Bonferroni correction on the significant level (i.e., 7 tests for 3 factors, *p* = 0.05/7 = 0.007), the analysis showed main effects of [Type, *F*(1,66) = 27.04, *p* < 0.001, η_p_^2^ = 0.29], [Delay, *F*(1,66) = 34.82, *p* < 0.001, η_p_^2^ = 0.51] and [Age, *F*(1,66) = 13.24, *p* < 0.001, η_p_^2^ = 0.17]. The interaction [Type * Delay, *F*(1,66) = 7.88, *p* < 0.001, η_p_^2^ = 0.19] was significant. The other interactions were not significant, [Type * Age, *F*(1,66) = 3.45, *p* = 0.068, η_p_^2^ = 0.05], [Delay * Age, *F*(1,66) = 1.20, *p* = 0.31, η_p_^2^ = 0.03], [Type * Delay * Age, *F*(1,66) < 1]. Post-hoc comparisons confirmed a benefit of the meaningful condition when memory was assessed immediately, in both adults (*t* = 3.43, *P*_*holm*_ = 0.041) and children (*t* = 4.84, *P*_*holm*_ < 0.001). After three weeks, this benefit however disappeared in both adults (*t* = 1.45, *P*_*holm*_ = 1), and children (*t* = 2.67, *P*_*holm*_ = 0.31). Again, there was no significant difference between the two types of images after six weeks, in both adults (*t* = − 0.79, *P*_*holm*_ = 1) and children (*t* = 0.187, *P*_*holm*_ = 1). To sum up, the results did not support the hypothesis that semantic information determines most of what remains in LTM^28^. Indeed, after 6-weeks, there was no evidence of a benefit for the meaningful images as compared to the meaningless images in both adults and children, at least, when they were presented twice for 1920 ms.

### False alarms for the meaningful images

**Figure 4:**
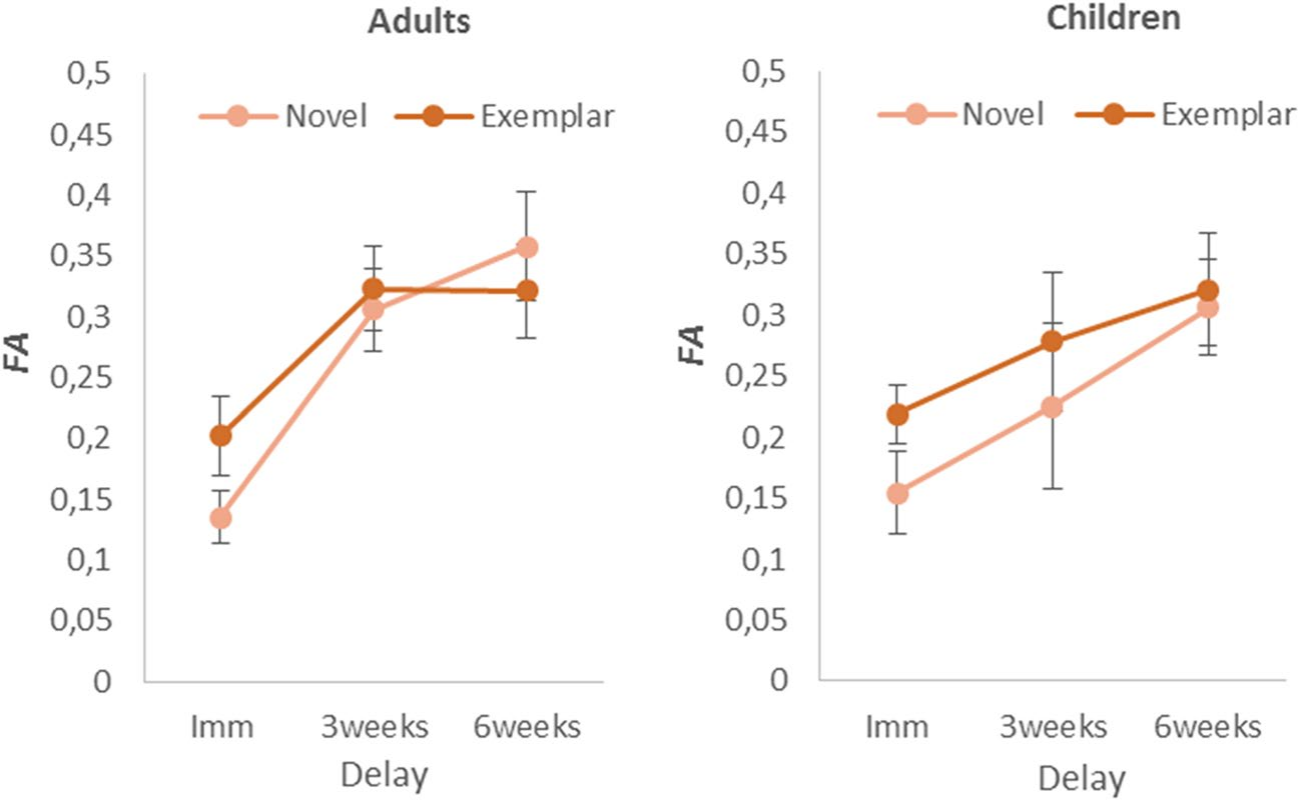
False alarms rates in the meaningful conditions “Novel” and “Exemplar” depending on the delay, for both adults (panel on the left) and children (panel on the right). The error bars show the standard error of the mean (n = 12).

The false alarms obtained from the meaningful images are shown in Fig. 4. Recall that the novel images belonged to a basic-level category that was not used during the learning phase, whereas the exemplar lures belonged to a basic-level category that had already been used during the learning phase (see Fig. 2).

To examine memory distortion regarding the new images that belonged to a category used in the learning phase (Exemplar condition), a mixed-design ANOVA was conducted on FA with Age (adults and children) and Delay (immediate, 3-weeks, and 6-weeks) as between-subject factors, and Type of images (novel vs. exemplar) as within-subject factors. The analysis revealed a main effect of Delay, *F*(2,66) = 7.50, *p* < 0.001, η_p_^2^ = 0.18 and Type, *F*(1,66) = 8.38, *p* < 0.001, η_p_^2^ = 0.11. There was no effect of Age, *F*(1,66) < 1, η_p_^2^ < 0.01. After Bonferroni correction on the significant level (*p* = 0.05/7 = 0.007), the interaction [Type * Delay, *F*(2,66) = 4.70, *p* = 0.012, η_p_^2^ = 0.12] and [Type * Age, *F*(2,66) = 1.93, *p* = 0.17, η_p_^2^ = 0.03] were not significant (for the two others interactions, *Fs* < 1). However, a Bayesian repeated measures ANOVA conducted with the variables Age, Delay and Type suggested that the best model was [Type + Delay + Type * Delay (BF_M_ = 9.38)], with evidence for the interaction between Type * Delay (BFincl = 7.27). This suggests that our correction for family-wise errors in the classical ANOVA might have been too conservative. Therefore, we examined the difference between false alarms in both Type conditions, with post-hoc comparisons conducted for the factors Type and Delay. We did not include the factor Age because both the classical and Bayesian analyses showed that this factor had no impact on false alarms. Those tests indicated that the false alarms were higher for the exemplar lures when memory was assessed immediately [mean diff. = 0.07%, *t* = 3.68, *P*_*holm*_ < 0.01]. The difference between the two types of images (novel vs. exemplar) was neither reliable at 3-weeks, [mean diff. = 0.03%, *t* = 1.95, *P*_*holm*_ = 0.39], nor reliable at 6-weeks, [mean diff. = − 0.01%, *t* = 0.62, *P*_*holm*_ = 1].

To summarize, although exemplar lures triggered more false alarms than novel images when memory was tested immediately after learning, the results suggest that this effect disappeared across weeks. Remember that the hypothesis that semantic information is extracted earlier and determines most of what remains in LTM^28^ leads to the prediction that False recognition for the exemplar lures should have been more numerous than false recognition for novel images, and that this effect should have increased over time. The present results lead to accept the first prediction but to reject the second one, i.e. the false alarms did not increase more across weeks for exemplar lures than for novel images (i.e. images that belonged to a basic-level category not used during the learning phase).

## General discussion

The purpose of the present study was to provide insight regarding the format and the content of the representations of pictures in visual LTM. More specifically, by examining how memory for meaningless and meaningful images evolved across weeks, we tested the hypothesis that conceptual information is extracted earlier and determines most of what remains in LTM^25–27^. Because the literature on visual memory used to report memory performance on both very brief and longer exposures to stimuli, the images were presented for either 120 ms or 1920 ms. Moreover, because we expected that a single exposure may be not enough to maintain an image across weeks in memory, the images were presented either once or twice. The hypothesis we examined leads to four predictions: (1) For very brief exposures, only meaningful images should be accessible to recognition; (2) Meaningless images should be more forgotten over weeks than meaningful images; (3) False recognition for the exemplar lures should be more numerous than false recognition for novel images, especially after weeks; (4) although speculative, we expected similar patterns for both children and adults.

In line with the first prediction, for 120 ms exposures, the recognition indexes (*d*_*a*_) were much better for the meaningful images than for the meaningless images. This confirms that indeed, for brief exposures, semantic information considerably enhances recognition memory^27,28,41^. Nonetheless, whether the images were meaningless or meaningful, they tended to be dramatically forgotten over weeks. Although a second exposure enhanced memory and then reduced the decay for meaningful images, it seems that two brief exposures are not sufficient to maintain a memory for a very long term. This decay was even more pronounced for the children than for the adults, quickly reaching chance level. Our results nevertheless contrast with the RSVP literature suggesting that with presentations shorter than around 250 ms, only the gist is retained in LTM^29^. Indeed, the performance in the meaningless condition was above chance level when the testing phase was presented immediately after learning or three weeks later, showing that 120 ms of exposure is sufficient to maintain much more than the gist in LTM, at least in adults. It is also noteworthy that in preliminary experiments using a similar procedure, we even observed a learning effect for meaningless images presented for only 30 ms (see also^35,36^).

The second aspect of the results concerns memory for longer exposures. Again, the results show a strong benefit for the meaningful images when memory was assessed immediately after learning. They also show how a second exposure considerably enhances recognition memory and delays the decay in memory. Furthermore, there was indeed a reliable interaction between the factor Delay (Immediate, 3-weeks and 6 weeks) and Type (meaningful vs. meaningless), but of particular interest, this interaction was in the opposite direction to what we predicted^29^. As a result, at six weeks, there was no longer any benefit for the meaningful images presented twice as compared to the meaningless images, suggesting that the semantic facilitation disappeared over weeks. This pattern of results was observed in both adults and children. This thus fails to validate the prediction that meaningless images should be forgotten more easily over weeks than meaningful images. Unpublished experiments conducted in our laboratory revealed a similar pattern of results with four-year-old children exposed to an abbreviated version of the materials, as well as when adults had to provide a label to the images during the learning phase.

The third prediction was related to the false alarms for the meaningful “exemplar lures” with respect to the meaningful “novel-gist” images. When the recognition took place immediately after learning, the false recognition for the exemplar lures (i.e., the images that belonged to a basic-level category already used in the learning phase) was above the false recognition observed with novel categories (novel images). Similar patterns were observed in both adults and children. This suggests that, indeed, gist is used in the retrieval of memory when it was assessed immediately after learning. However, this effect disappeared after three weeks. Again, this result goes against to our initial prediction.

The last prediction was related to the effects of age on images memory. In line with our initial prediction, memory performance was weaker in nine-year-old children than in adults. Moreover, the global pattern was similar, despite a rapid floor effects in children memory for the images presented briefly. The children’s capacity to form and retrieve visual representations nevertheless suggest the existence of a visual memory system that might be similar to the visual memory system of adults. As mentioned above, children were even more inclined to forget images presented very briefly than adults. Several reasons are likely to explain this result. This might be the signature of a kind of immaturity of the attentional, working memory, or declarative memory systems. It can be noted that they also had much more difficulty using all the panel of the confidence scale. A simpler scale or a two-alternative forced choice task could be more appropriate for a young population.

Together, the results obtained in the present study call into question the models of VLTM for images that assume that conceptual information determines most of what remains in LTM, e.g.^25,26,28,28,42^. Though conceptual/semantic information and even linguistic labels enhance the encoding & maintaining of representations in LTM considerably, through a dual-coding for example^6,7^, semantic codes or even the gist might not be what remains primarily in LTM over weeks. By contrast, VLTM has a strong capacity to store visual features of images, even independently of pre-existing conceptual features, provided that the exposure is long enough and repeated. In addition, memory for visual information contained in images seems to be more robust over time than memory for semantic information that would be independent of visual features, as suggested by the result that the false alarms did not increase more in the exemplar lure condition than in the novel condition.

However, this study shows also that interfering effects and false memories constitute a problem when investigating recognition memory^43^. In congruence with the literature on *memory distortion*, false alarms were higher in the meaningless condition when memory was tested immediately after learning, but this effect tended to reverse over weeks. As a result, the stronger impact of the delay on the *d*_*a*_ in the meaningful condition as compared to the meaningless condition (for extended and multiple exposures) was not due to a stronger impact on the hits (i.e. impact on decay) but to a stronger impact on the false alarms (i.e. impact on interference).

The present results raise several questions. First, what makes an image memorable over time^22,44^? This study shows a potentiating effect of repetition and exposure duration on memory over weeks, and suggests that multiple and extended exposures are probably required to maintain an image in LTM over time. Second, the present results highlight important changes in memory effects over weeks, with a reduction of the facilitating effect of the meaningful cues in the repeated and prolonged exposure condition. Thus, we hypothesize that multiple and prolonged exposures, the uniqueness of an image, as well as its distinctiveness relative to what is already in memory are good predictors of which images will be sensitive to long term recognition^45^. Note that other factors, such as emotional valence regarding the stimulus, or attentional resource allocated to the stimulus play probably a crucial role as well. Nevertheless, the present study provides an argument to the thesis that the coding of images in very long term memory might be based more on visual features than on semantic codes.

Second, the present study raises the question of how the different kinds of consolidation mechanisms (synaptic vs systemic), as well as how the different memory/processing systems interact during the encoding and the consolidation of visual memories^46,47^. General theories of memory (e.g.^48–50^) used to propose a distinction between explicit/declarative and implicit/nondeclarative memory systems. In this respect, a hypothetical sketch is that memory of images results from interactions between different memory systems. An “integrating system”, usually associated with explicit/declarative memory, might play a critical role in the integration and association of distributed sensory and conceptual information. The hippocampus might be a good candidate for such integration and memory formation. This system would underlie VLTM that is strongly enhanced by the retrieving of semantic cues. However, the associations maintained in this system would rapidly decay over weeks because of important neuronal recycling. In parallel, learning mechanisms relying on the mere extraction of visual information would develop at a lower level of visual processing. Information coded by this system is visual by nature. Such mechanisms require both longer and multiple exposures to a specific stimulus to support familiarity, but would be more robust over time and less subject to interference effects. Pervasive cortical plasticity phenomena (e.g. Spike Timing Dependent Plasticity) are good candidates to account for the formation of such sensory memories^51,52^.

However, research conducted in the fields of implicit learning and statistical learning reveal the limits of such a clear functional dichotomy between explicit and implicit memory systems that would be governed by different learning principles and that would operate in isolation from each other. Memory phenomena result in large part from both external, slow, pervasive, and cortex-based mechanisms of learning, and on transitory associative representations formed and maintained within the medial temporal lobe memory system^46,53–55^. In addition, important changes in the functional connectivity between the hippocampus and cortical areas operate during memory consolidation, especially with a progressive disengagement of the Medial Temporal Lobe and both synaptic and systemic consolidation in the neocortex^47,56,57^. How those different memory systems interact and how a redescription of knowledge operates over time and consolidation remain a challenge for further research. In this view, the present research highlights important changes in memory across weeks, which show its relevance for assessing memory after weeks and months. The weakness of most research in the field of visual LTM is that it examines memory immediately after learning only. Studying how memories evolve over time remains fundamental to understand the format and the content of memories in LTM.

To conclude, the present study shows that while semantic information enhances learning of images in LTM systems for transitory periods, they might not be able to account for memorization of images in the long term. In contrast, information stored at a lower level might be more robust over time and might be more resistant to interfering effects. This hypothesis could be examined by assessing memory over months or even years for images that are presented several times. The problem of how images are stored and manipulated within the human brain remains a fertile area for further research and to address the issue of the coding of information into memory.

## Method

Participants: Thirty-six adult individuals (mean age = 26 years; SD=6 years, range = 17-42 years) participated and thirty-six 9 year-old children participated in the experiment. All were naïve to the purpose to the study and reported normal or corrected-to-normal acuity with no color vision deficiencies. The adult participants received course credits and gave written informed consent before starting the experiment. The parents of the children signed a similar informed consent form. The children were free to accept or to refuse participation in the experiment both for the learning phase and for the testing phase. The procedures were in accordance with the Declaration of Helsinki and approved by the local ethics committee “Comité d’Evaluation Ethique de l’Inserm”.

Material: The material included 360 different full-colored images, with 200 “meaningful” images and 160 “meaningless” images (for several examples, see Fig. 1). An additional 8 images (4 meaningless and 4 meaningful) were used for a practice block of learning. The images came from the CerCo lab’s collection of images.

*Procedure* The experiment included two phases: a learning phase followed by a testing phase.

*Learning phase* In the learning phase, observers were presented with 200 different full-colored images, of which 80 were meaningless and 120 were meaningful. The 120 meaningful images were photographs of either an animal, a vegetal, an object or a landscape. The signified/gist represented in each picture belonged to a unique basic-level category and was chosen because it could be quickly labeled using a simple name (e.g. a dog, a cherry, a beach).

**Figure 5:**
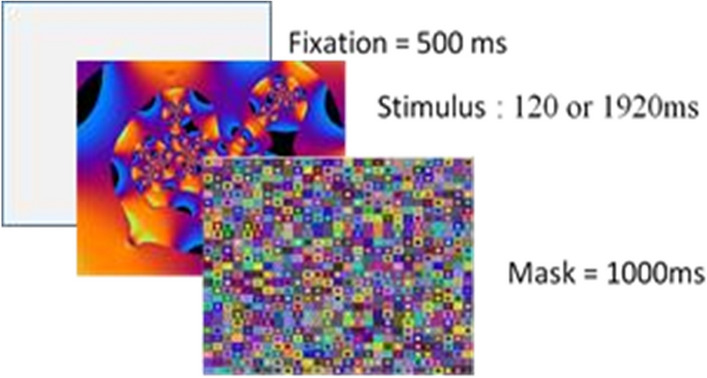
Sequence of a trial during the learning phase. Each trial started by a 500-ms cross fixation, followed by an image, then by a 1000-ms complex mask. The images came from the CerCo lab’s collection of images.

Among the 80 meaningless images, 20 images were presented once during 120 ms, 20 were presented twice during 120 ms, 20 were presented once during 1920 ms and 20 were presented twice during 1920 ms. Among the 120 meaningful images, 30 were presented once during 120 ms, 30 were presented twice during 120 ms, 30 were presented once during 1920 ms and 30 were presented twice during 1920 ms. This gave a total of 300 trials. Note that the additional 40 meaningful images (10 in each of the four exposure conditions) were used to create the “Exemplar” condition in the recognition task (description in the paragraph “testing phase”). Each trial started by a 500-ms cross fixation, followed by an image, then by a 1000-ms complex mask (for an example, see Fig. 5).

The participants were instructed to remember each image as well as possible for a further memory task. They additionally performed a repetition detection task to maintain focus. They were told to press a button to indicate if the current item had been presented previously. The learning phase began after 12 familiarization trials that included four repeated images. After this familiarization, an instruction indicated the beginning of the experiment. The participants were exposed to the 300 trials (100 images presented once and 100 images presented twice). The order of presentation of the images, and consequently, the exposure duration and the number of repetitions of the images were all randomized across the experiment. Every 30 trials, the participants were shown a screen allowing them to take a break. They were free to continue the experiment when they were ready by pressing the space bar. The exposure duration, as well as the number of exposures for each image were counterbalanced between the participants.

*Testing phase* Participants were split into three different “delay groups” (12 per condition), in such a way that the testing phase was either administrated immediately after the learning phase, three weeks later or six weeks later. The memory of the participants for the images was assessed in a recognition task. Observers were presented with 360 images, that is, the 80 meaningless images that were presented in the learning phase (Meaningless-Old condition), 80 new meaningless images that were never seen before (Meaningless-New condition), 80 meaningful images from the 120 that were presented during the learning phase (Meaningful-Old condition), and 80 new meaningful images that were never seen before. Among the 80 new meaningful images, 40 belonged to 40 basic-level categories that were not used during the learning phase (Novel condition), and 40 belonged to 40 basic-level categories that were already used during the learning phase (Exemplar condition, for an example, *see *Fig. 2). Each image was displayed for 3s. The participants were asked to decide whether or not they had seen the image in the study phase. Then, they rated the confidence in their response on a scale from 1 to 4. The scale was presented as follows: “Confidence? 1= just guessing, 2 = not sure, 3 = confident, 4 = very sure. The images that were used in the new conditions vs. the images that were used in the old conditions were counterbalanced between the participants.

The procedure of the experiment was programmed on Python and the stimuli were implemented with Open Sesame. The data were analyzed with Jasp.

## Supplementary Information

Supplementary Information 1.

Supplementary Information 2.

## Data Availability

The datasets used and/or analyses during the current study are available from the corresponding author on request.

## References

[1] Pylyshyn ZW (2002). Mental imagery: In search of a theory. Behav. Brain Sci..

[2] Pylyshyn Z (2003). Return of the mental image: Are there really pictures in the brain?. Trends Cogn. Sci..

[3] Borst G, Kosslyn SM, Denis M (2006). Different cognitive processes in two image-scanning paradigms. Mem. Cogn..

[4] Kosslyn SM (1975). Information representation in visual images. Cogn. Psychol..

[5] Shepard RN, Metzler J (1971). Mental rotation of three-dimensional objects. Science (80-.).

[6] Clark JM, Paivio A (1991). Dual coding theory and education. Educ. Psychol. Rev..

[7] Paivio A (1986). Imagery and Verbal Processes.

[8] Shepard RN (1967). Recognition memory for words, sentences, and pictures. J. Verbal Learn. Verbal Behav..

[9] Standing L (1973). Learning 10000 pictures. Q. J. Exp. Psychol..

[10] Nelson DL (1979). ‘Remembering pictures and words: Appearance, significance and name. Inf. Process. Res. Advert..

[11] Simons DJ, Levin DT (1997). Change blindness. Trends Cogn. Sci..

[12] Intraub H (1981). Rapid conceptual identification of sequentially presented pictures. J. Exp. Psychol. Hum. Percept. Perform..

[13] Irwin DE (1992). Memory for position and identity across eye movements. J. Exp. Psychol. Learn. Mem. Cognit..

[14] O’Regan JK, Noë A (2001). A sensorimotor account of vision and visual consciousness. Behav. Brain Sci..

[15] Rensink RA (2000). The dynamic representation of scenes. Vis. Cogn..

[16] Simons DJ (1996). In sight, out of mind: When object representations fail. Psychol. Sci..

[17] Rensink RA, Kevin OJ, Clark JJ (2000). On the failure to detect changes in scenes across brief interruptions. Vis. Cogn..

[18] Hollingworth A, Henderson JM (2002). Accurate visual memory for previously attended objects in natural scenes. J. Exp. Psychol. Hum. Percept. Perform..

[19] Brady TF, Konkle T, Alvarez GA (2011). A review of visual memory capacity: Beyond individual items and toward structured representations. J. Vis..

[20] Schurgin MW (2018). Visual memory, the long and the short of it: A review of visual working memory and long-term memory. Atten. Percept. Psychophys..

[21] Brady TF, Konkle T, Alvarez GA, Oliva A (2008). Visual long-term memory has a massive storage capacity for object details. Proc. Natl. Acad. Sci..

[22] Isola P, Xiao J, Parikh D, Torralba A, Oliva A (2014). What makes a photograph memorable?. IEEE Trans. Pattern Anal. Mach. Intell..

[23] Isola P, Xiao J, Torralba A, Oliva A (2011). What makes an image memorable?. Proc. IEEE Comput. Soc. Conf. Comput. Vis. Pattern Recognit..

[24] Rust NC, Mehrpour V (2020). Understanding image memorability. Trends Cogn. Sci..

[25] Brady TF, Alvarez GA, Störmer VS (2019). The role of meaning in visual memory: Face-selective brain activity predicts memory for ambiguous face stimuli. J. Neurosci..

[26] Cunningham CA, Yassa MA, Egeth HE (2015). Massive memory revisited: Limitations on storage capacity for object details in visual long-term memory. Learn. Mem..

[27] Konkle T, Brady TF, Alvarez GA, Oliva A (2010). Scene memory is more detailed than you think: The role of categories in visual long-term memory. Psychol. Sci..

[28] Potter MC (2012). Conceptual short term memory in perception and thought. Front. Psychol..

[29] Potter MC (2012). Recognition and memory for briefly presented scenes. Front. Psychol..

[30] Gathercole SE (1999). Cognitive approaches to the development of short-term memory. Trends Cogn. Sci..

[31] Brainerd CJ, Holliday RE, Reyna VF (2004). Behavioral measurement of remembering phenomenologies: So simple a child can do it. Child Dev..

[32] Richmond J, Nelson CA (2007). Accounting for change in declarative memory: A cognitive neuroscience perspective. Dev. Rev..

[33] Rose SA, Feldman JF, Jankowski JJ (2004). Infant visual recognition memory. Dev. Rev..

[34] Ferrara K, Furlong S, Park S, Landau B (2017). Detailed visual memory capacity is present early in childhood. Open Mind.

[35] Thunell E, Thorpe SJ (2019). Memory for repeated images in rapid-serial-visual-presentation streams of thousands of images. Psychol. Sci..

[36] Thunell E, Thorpe SJ (2019). Regularity is not a key factor for encoding repetition in rapid image streams. Sci. Rep..

[37] Brady TF, Konkle T, Oliva A, Alvarez GA (2009). Detecting changes in real-world objects: The relationship between visual long-term memory and change blindness. Commun. Integr. Biol..

[38] Rotello CM, Masson MEJ, Verde MF (2008). Type I error rates and power analyses for single-point sensitivity measures. Percept. Psychophys..

[39] Yonelinas AP (1994). Receiver-operating characteristics in recognition memory: Evidence for a dual-process model. J. Exp. Psychol. Learn. Mem. Cogn..

[40] Cramer AOJ (2016). Hidden multiplicity in exploratory multiway ANOVA: Prevalence and remedies. Psychon. Bull. Rev..

[41] Kouststaal W (2003). False recognition of abstract versus common objects in older and younger adults: Testing the semantic categorization account. J. Exp. Psychol. Learn. Mem. Cogn..

[42] Konkle T, Brady TF, Alvarez GA, Oliva A (2010). Categories in visual long-term. Memory..

[43] Schacter DL, Guerin SA, Jacques PLS (2011). Memory distortion: An adaptive perspective. Trends Cogn. Sci..

[44] Khosla A, Raju AS, Torralba A, Oliva A (2015). Understanding and predicting image memorability at a large scale. Proc. IEEE Int. Conf. Comput. Vis..

[45] Brown GDA, Neath I, Chater N (2007). A temporal ratio model of memory. Psychol. Rev..

[46] Goujon A, Didierjean A, Thorpe S (2015). Investigating implicit statistical learning mechanisms through contextual cueing. Trends Cogn. Sci..

[47] Larzabal C, Bacon-Macé N, Muratot S, Thorpe SJ (2020). Tracking your mind’s eye during recollection: Decoding the long-term recall of short audiovisual clips. J. Cogn. Neurosci..

[48] Reber PJ, Squire LR (1994). Parallel brain systems for learning with and without awareness. Learn. Mem..

[49] Squire LR (1992). Declarative and nondeclarative memory: Multiple brain systems supporting learning and memory. J. Cogn. Neurosci..

[50] Tulving E (1972). Episodic and semantic memory. Organ. Mem..

[51] Markram H, Gerstner W, Sjöström PJ (2012). Spike-timing-dependent plasticity: A comprehensive overview. Front. Synaptic Neurosci..

[52] Masquelier T, Guyonneau R, Thorpe SJ (2009). Competitive STDP-based spike pattern learning. Neural Comput..

[53] Goujon A, Didierjean A, Poulet S (2014). The emergence of explicit knowledge from implicit learning. Mem. Cogn..

[54] Henke K (2010). A model for memory systems based on processing modes rather than consciousness. Nat. Rev. Neurosci..

[55] Henke K, Reber TP, Duss SB (2013). Integrating events across levels of consciousness. Front. Behav. Neurosci..

[56] Frankland PW, Bontempi B (2005). The organization of recent and remote memories. Nat. Rev. Neurosci..

[57] Takeda M (2019). Brain mechanisms of visual long-term memory retrieval in primates. Neurosci. Res..

