# Supplementary material for: The fate of visual long term memories for images across weeks in adults and children
Source: Sci Rep. 2022 Dec 16;12:21763. doi: 10.1038/s41598-022-26002-7 (PMC9758234; doi:10.1038/s41598-022-26002-7)
Supplement: Supplementary file 2 — Supplementary Information 2. [file 41598_2022_26002_MOESM2_ESM.docx]

The Fate of Visual Long Term Memories for Images across Weeks in Adults and Children

Annabelle Goujon

*Université de Franche-Comté*

Fabien Mathy

*BCL-CNRS & Université de Côte d’Azur*

Simon Thorpe

*CerCo-CNRS & Université de Toulouse 3*

Please address correspondence to:

Annabelle Goujon

annabelle.goujon@univ-fcomte.fr

Laboratoire de Recherches Intégratives en Neurosciences et Psychologie Cognitive
UFR Santé, bâtiment Rabelais
19 rue Ambroise Paré - 25030 Besançon, Cedex, France

+33(0)3-81-66-54-41 - fax : 0+33(0)3-81-66-54-40

# Supplementary materials

***Tables of mean results and figures representing ROC curves***

|  | **Condition** | **Exposure duration** | **Exposures number** | **Immediate delay** | **Three-weeks delay** | **Six-weeks delay** |
| --- | --- | --- | --- | --- | --- | --- |
| **Hits** | Meaningful-Old | 120  120  1920  1920 | 1  2  1  2 | 0,650 (0,056)  0,775 (0,043)  0,879 (0,035)  0,971 (0,018) | 0,392 (0,041)  0,617 (0,047)  0,674 (0,037)  0,888 (0,029) | 0,485 (0,053)  0,629 (0,042)  0,608 (0,037)  0,804 (0,038) |
|  | Meaningless-Old | 120  120  1920  1920 | 1  2  1  2 | 0,392 (0,051)  0,521 (0,054)  0,733 (0,042)  0,908 (0,024) | 0,317 (0,022)  0,350 (0,046)  0,492 (0,030)  0,792 (0,044) | 0,221 (0,045)  0,259 (0,056)  0,350 (0,059)  0,725 (0,061) |
| **False alarms** | Meaningful-Exemplar |  |  | 0,202 (0,022) | 0,323 (0,034) | 0,321 (0,045) |
|  | Meaningful-Novel |  |  | 0,135 (0,032) | 0,306 (0,035) | 0,358 (0,039) |
|  | Meaningless-New |  |  | 0,181 (0,034) | 0,234 (0,026) | 0,232 (0,045) |

**Table S1: Hits as a function of type of images, exposure duration, number of exposures, and delay, as well as false alarms as a function of type of images observed, for adults in the recognition task. Parentheses represent standard errors.**

|  | **Condition** | **Exposure duration** | **Exposures number** | **Immediate delay** | **Three-weeks delay** | **Six-weeks delay** |
| --- | --- | --- | --- | --- | --- | --- |
| **Hits** | Meaningful-Old | 120  120  1920  1920 | 1  2  1  2 | 0,571 (0,070)  0,688 (0,059)  0,879 (0,037)  0,963 (0,016) | 0,318 (0,056)  0,375 (0,042)  0,460 (0,042)  0,648 (0,044) | 0,336 (0,055)  0,424 (0,050)  0,509 (0,058)  0,695 (0,060) |
|  | Meaningless-Old | 120  120  1920  1920 | 1  2  1  2 | 0,229 (0,041)  0,333 (0,070)  0,550 (0,070)  0,788 (0,056) | 0,156 (0,049)  0,200 (0,066)  0,211 (0,050)  0,423 (0,073) | 0,155 (0,038)  0,258 (0,056)  0,235 (0,041)  0,550 (0,056) |
| **False alarms** | Meaningful-Exemplar |  |  | 0.219 (0.034) | 0.278 (0.068) | 0.321 (0.039) |
|  | Meaningful-Novel |  |  | 0,154 (0,024) | 0,225 (0,057) | 0,306 (0,046) |
|  | Meaningless-New |  |  | 0,187 (0,041) | 0,157 (0,055) | 0,214 (0,042) |

**Table S2: Hits as a function of type of images, exposure duration, number of exposures, and delay, as well as false alarms as a function of type of images observed, for 9-year-old children in the recognition task. Parentheses represent standard errors.**

**Figure S1: Receiver-operating curves in adults for both types of images (meaningless: panels on the left and meaningful: panels on the right) and each condition (120ms-1exposure, 120ms-2exposures, 1920ms-1exposure, 1920ms-2exposures). Each point on the curve represents a different level of confidence (from 1 to 4: the images were classified as old (1= very sure; 4= just guessing); from 5 to 8: the images were classified as new (5=just guessing; 8=very sure). The point were calculated by cumulating response proportions of hits and false alarms, depending of the degree of response bias (1 to 8).**

**
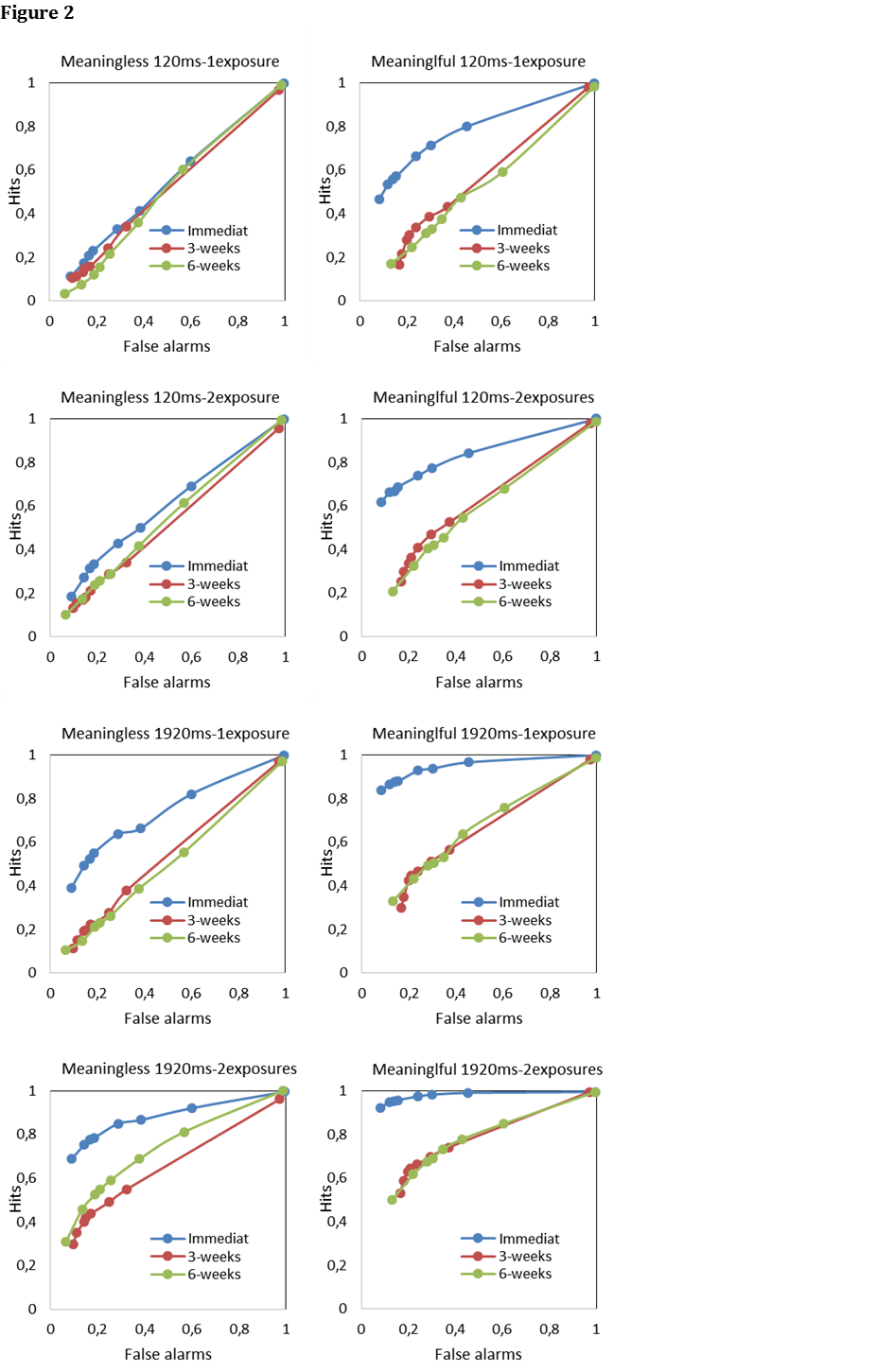
Figure S2: Receiver-operating curves in children for both types of images (meaningless: panels on the left and meaningful: panels on the right and each condition (120ms-1exposure, 120ms-2exposures, 1920ms-1exposure, 1920ms-2exposures).**

***Bayesian analyses on d_a_***

Bayesian analyses on the factor Age: Because the performance of children quickly reached floor effects when the images were presented once during only 120ms, there was no rational to include those data into the analyses. Therefore, we first ran a Bayesian repeated measures ANOVA on *d_a_* derived from the images presented twice, with the factors Age, Delay, Type and Duration. The result showed that the most parsimonious model was: Type + Duration + Age + Delay + Type * Duration + Type * Delay + Duration * Delay (BF _M_ = 40). Thus, there was no interaction with Age (all BFincl < 1.5). Note that this analysis confirmed a strong interaction Type * Delay (BFincl > 2500). A second Bayesian repeated measures ANOVA was conducted on the images presented for 1920ms. The result revealed that the most parsimonious model was: Type + Number + Age + Delay + Type * Number + Type * Age + Number * Age + Type * Delay (BF _M_ = 65). However, for both interactions with Age (i.e., Type * Age and Number * Age), the respective BF incl (< 1.5) suggested anecdotal effects. Therefore, both classical ANOVA and Bayesian ANOVA analyses were consistent: the pattern of results were not statistically different between children and adults. Again, the analysis confirmed a strong interaction Type * Delay (BFincl > 2500).

Bayesian analyses to confirm the effects revealed by the classical ANOVA: To examine the robustness of the effects revealed by the classical ANOVA, a Bayesian repeated measures ANOVA was run on all factors except the factor age. We removed the factor age as its relevance was discarded from the classical ANOVA and from the Bayesian analyses described above, but also to allow the model to be computable. The result indicated that the most parsimonious model was: Type + Number + Duration + Delay + Type * Number + Type * Duration + Number * Duration + Type * Delay + Duration * Delay + Type * Number * Duration (BF _M_ = 221).  Said otherwise, the only absent first-order interaction was Number * Delay and there was only one present second-order interaction (i.e., Type * Number * Duration). We obtained a BFincl = 13.9 showing strong evidence for this second order interaction. However, although included in the best model, the interactions Type * Number and Type * Duration lead to a BFincl < 3 and thus could be considered as an anecdotal effect. All other terms of the model had a BFincl superior to 2.27e9, showing extreme evidence for the respective effects of the best model. Therefore, the effects revealed by the Bayesian analyses were similar to those revealed by the classical ANOVA (when we considered a Bonferroni correction on p-values); and even though the factor Age was not included in the Bayesian analysis.
